# Organic Glycinate Trace Minerals Improve Hatchability, Bone and Eggshell Breaking Strength, and Mineral Uptake During Late Laying Cycle in Layer Breeders

**DOI:** 10.3390/vetsci12100927

**Published:** 2025-09-24

**Authors:** Mujtaba Akram Jahangir, Muhammad Muneeb, Muhammad Farooq Iqbal, Syeda Maryam Hussain, Syed Sohail Habib, Sohail Ahmad, Kasim Sakran Abass, Nasir Mukhtar, Rashed A. Alhotan, Ali R. Al Sulaiman, Ala E. Abudabos

**Affiliations:** 1 Department of Livestock Production and Management, Faculty of Veterinary and Animal Sciences, PMAS Arid Agriculture University, Rawalpindi 46300, Pakistan; mujtabajahangir1@gmail.com (M.A.J.); syedamaryam@uaar.edu.pk (S.M.H.); nmukhtar@uaar.edu.pk (N.M.); 2 Department of Animal Nutrition, Faculty of Animal Production and Technology, University of Veterinary and Animal Sciences, Lahore 54000, Pakistan; 2022-mphil-1331@uvas.edu.pk; 3 Department of Poultry Production, Faculty of Animal Production and Technology, University of Veterinary and Animal Sciences, Lahore 54000, Pakistan; sohail.ahmad@uvas.edu.pk; 4 Department of Physiology, Biochemistry, and Pharmacology, College of Veterinary Medicine, University of Kirkuk, Kirkuk 36001, Iraq; kasim_abass@uokirkuk.edu.iq; 5 Department of Animal Production, College of Food and Agriculture Sciences, King Saud University, P.O. Box 2460, Riyadh 11451, Saudi Arabia; ralhotan@ksu.edu.sa; 6 Environmental Protection Technologies Institute, Sustainability and Environment Sector, King Abdulaziz City for Science and Technology, P.O. Box 6086, Riyadh 11442, Saudi Arabia; arsuliman@kacst.gov.sa; 7 Department of Food and Animal Sciences, College of Agriculture, Tennessee State University, Nashville, TN 37209, USA

**Keywords:** trace minerals, layer breeder, reproductive performance, egg quality, mineral excretion

## Abstract

This study evaluated the effects of replacing inorganic trace minerals with organic glycinate forms at two inclusion levels in layer breeders during the late laying cycle over 6 weeks (66 to 72 weeks of age). For this, a total of 180 layer breeders (162 hens and 18 males; Lohmann LSL Ultralite) were randomly divided into three treatment groups having six replicates of nine hens each and one male for each replicate following a completely randomized design (CRD). The dietary treatments included: (1) Basal diet + inorganic trace minerals at breed recommended levels (ITM100), (2) Basal diet + organic trace minerals at recommended levels (OTM100), (3) Basal diet + organic trace minerals at half dose of recommended levels (OTM50). The trace mineral contents in the samples (feed, bone, and excreta) were determined through inductively coupled plasma optical emission spectroscopy. The results indicated that glycinate minerals supplementation at half dose enhanced (*p* ≤ 0.05) tibial breaking strength compared to the inorganic minerals (38.97 vs. 29.55 MPa). Similarly, egg quality (egg geometry, yolk index, eggshell properties), and hatching traits (hatchability and fertility) were enhanced (*p* ≤ 0.05) following the use of glycinate organic minerals as compared to inorganic minerals. Copper deposition was higher (*p* ≤ 0.05) in the OTM100 as compared to other groups. Excreta levels of zinc (Zn) and manganese (Mn) were higher (*p* ≤ 0.05) in the ITM100 group compared to OTM100 and OTM50. In conclusion, supplementation of glycinate trace organic minerals to layer breeder hens during the late laying cycle had enhanced the bone and eggshell breaking strength, hatching traits, and absorption of certain trace minerals.

## Simple Summary

This study assessed the effects of substituting inorganic trace elements with organic glycinate forms on the performance of layer breeder hens during the late laying cycle. A total of 180 birds were allocated into three treatments: one group received inorganic minerals at the required levels, while the other two groups received glycinate trace minerals at full and half of the standard amounts, respectively. The findings suggested that birds receiving glycinate minerals had superior egg quality, bone strength, fertility, and hatchability compared to those fed inorganic minerals. Moreover, organic mineral supplementation resulted in reduced fecal mineral excretion and enhanced copper deposition. These data imply that organic glycinate minerals may improve breeder performance and mineral absorption in chickens at the late stage of the production cycle.

## 1. Introduction

Layer breeders, the cornerstone of the commercial egg industry, serve as the parent stock for laying hens that cater to the growing global demand for table eggs. Layer breeders represent the industry’s commitment to ensuring a consistent supply of this essential food source worldwide [1]. The success of layer breeder management relies on multiple factors, with nutrition playing a central role in supporting optimal health, reproductive performance, and egg quality, which ultimately determine the productivity and longevity of the flock [2]. To address these challenges, poultry nutritionists continually strive to improve the productivity and health of aging breeder birds. Cracked and broken eggs result in economic losses ranging from 8% to 11% of total egg production across all stages of the supply chain [3]. Mineral nutrition, particularly the supplementation of organic glycinate trace minerals, plays a crucial role in regulating numerous physiological functions essential for productivity [4]. Among essential nutrients, trace minerals such as zinc (Zn), manganese (Mn), iron (Fe), and copper (Cu) are essential due to their involvement in key physiological processes that maintain bird health and egg production [5]. The sources and supplementation level of these trace minerals are critical in diet formulation for breeder birds, especially during the late laying cycle, as they significantly influence eggshell quality, skeletal integrity, and overall health [6].

Traditionally, inorganic mineral sources, such as sulfates and oxides, have been used in poultry feed formulations; however, these molecules are prone to dissociation in the gut, leading to lower bioavailability for birds [7]. To overcome this low bioavailability, nutritionists typically increase the inclusion levels of inorganic trace minerals in poultry diets. However, excessive supplementation of inorganic minerals can result in mineral antagonism, saturation of metal-binding proteins in the gut, and the accumulation of free ions. These free ions raise the possibility of mineral toxicity, cause oxidative tissue damage, and increase the amount of minerals excreted into the environment [8]. These challenges highlight difficulties with the optimization of genetic potential, environmental contamination, and intricate mineral interactions in commercial chicken farming [9].

To address these issues, the chicken industry has progressively transitioned to organic chelated mineral sources. In the avian digestive system, organic minerals that are attached to proteins, peptides, or amino acids improve mineral stability, absorption, and utilization [10]. Organic minerals have been reported to enhance nutrient absorption and safeguard minerals from antagonistic interactions, including binding by phytic acid and other competing substances in the gastrointestinal tract. Moreover, organic minerals have been documented to improve eggshell quality, fortify bones, bolster immunological function, and diminish environmental pollution from mineral excretion [11].

Although existing literature endorses the utilization of organic chelated minerals in poultry [12,13,14,15], important knowledge gaps remain—particularly regarding their application in aging layer breeders during the late laying phase, a period marked by declining egg quality, reduced mineral absorption, and increased skeletal fragility. Most existing research has focused on the use of organic minerals in broilers or young laying hens, with limited attention to breeder hens in their late production stage, where the nutritional demands are significantly different and more challenging [16,17]. Additionally, the optimal inclusion levels of organic trace minerals, especially in the form of glycinate chelates, remain poorly defined, and the comparative effects of replacement of inorganic minerals with their organic counterparts on key physiological and reproductive parameters have not been thoroughly investigated. Therefore, this study investigates the effects of substituting inorganic minerals (zinc, manganese, iron, and copper) with their respective glycinate complexes at 50% and 100% of the breed-recommended levels in the diets of layer breeders during the late laying cycle on laying performance, bone breaking strength, egg quality parameters, and mineral excretion. The hypothesis posits that replacing the inorganic trace minerals with organic glycinate forms at standard or even half the dose recommended by the strain guidelines would offer better performance in promoting egg quality, bone breaking strength, reproductive performance, and reducing mineral excretion in layer breeders during the late laying cycle.

## 2. Materials and Methods

### 2.1. Ethical Consent

This study was conducted at the Avian Research and Teaching Station (ARTS), Department of Poultry Sciences, Pir Mehr Ali Shah (PMAS), Arid Agriculture University, Rawalpindi, Pakistan. The study received official approval from the Institutional Ethics Committee of the university through notification (PMAS/DAS/481; Dated: 31 May 2024). The research trial was performed on layer breeders (Lohmann LSL Ultralite White) kept in cages over 6 weeks during the late laying cycle (66 to 72 weeks of age). A 14-day (2 weeks) adjustment period was provided before the commencement of the trial.

### 2.2. Experiment Design

A total of 180 layer breeders (162 hens and 18 males) were randomly divided into three treatment groups; each having six replicates of nine hens and one male each, following a completely randomized design. The birds were randomly assigned to three dietary treatments: one control group and two experimental groups. The control group (ITM100) received diets containing 100% inorganic form of the selected trace minerals. In the experimental groups, inorganic trace minerals were replaced with their organic forms: 100% replacement (OTM100) and 50% replacement (OTM50), based on the breed-recommended levels (Table 1). Glycinate chelates (E.C.O Trace^®^, Biochem mbH, Oldenburg, Germany) of the selected trace minerals were used as an organic source for the current study. The E.C.O. Trace^®^ mineral premix contained four trace minerals (Zinc ≥ 25%, Copper ≥ 23% Manganese ≥ 20%, and Iron ≥ 20%).

### 2.3. Bird Husbandry

The birds were housed under standard management conditions in an environmentally controlled cage system, with 16 h of light per day and relative humidity maintained at 65%. The birds were kept in conventional breeder cages constructed with galvanized wire; each replicate included nine hens, while one male was allocated separately and used to inseminate the hens by artificial insemination, as per the treatment design. The cage dimensions were 120 cm × 60 cm × 45 cm (length × width × height), and 800 cm^2^ (0.85 square feet) per bird was allocated. Each cage was equipped with a linear feeder, nipple drinkers, and plastic egg trays to ensure easy access to feed, water, and egg collection, and to maintain biosecurity and cleanliness throughout the trial period.

Diets based on corn and soybean meal (SBM) were formulated for all treatment groups. Birds were manually offered up to 110 g of feed per day in accordance with Lohmann guidelines. Basal diets were formulated according to the breed standards. The detailed dietary formulations and mineral inclusion levels are presented in Table 2. Moreover, the diet of males was also formulated following the strain recommendations [18].

The artificial insemination (AI) procedure was followed to ensure the successful fertilization of the breeding hens. Artificial insemination was performed every fourth day throughout the study. Insemination equipment, including guns and syringes, was thoroughly cleaned and sterilized. Semen was collected from the male birds through the massage method [19] and extended using a locally available semen extender to maintain its viability [20]. The hens were gently restrained, and their cloacal area was cleaned before insemination. A lubricated insemination tube was carefully inserted into the vent, and semen was slowly deposited into the reproductive tract [18].

### 2.4. Egg Quality and Shell Breaking Strength

Fresh eggs were collected daily from the experimental facility, and the eggs exhibiting visible deformities, cracks, or abnormalities were excluded. For egg quality attributes, five eggs per replicate (n = 30/treatment) were collected on the 40th day of the experimental period. The egg geometry (egg weight, egg length, egg breadth, and egg shape index) and internal egg quality indices (albumen weight, albumen height, yolk weight, yolk diameter, yolk height, and yolk index) were determined according to earlier reports [21,22,23]. Haugh units were determined as previously outlined [24].

Haugh Units (HU) = 100 × log_10_ (AH − 1.7 EW^0.37^ + 7.6).

where AH is albumen height in mm, and EW is egg weight in grams.

On the 40th day, a total of 90 eggs were selected for evaluation of eggshell strength, with 30 eggs from each treatment and five from each replicate. Eggshell strength was measured using an egg force reader (FGV-10XY, Nidec-Shimpo, Kyoto, Japan) as previously described [25,26,27]. Briefly, the device was calibrated before testing to ensure accuracy. Each egg was positioned vertically on the test platform, with the broader end facing downwards. A progressively increasing force was applied until the shell ruptured, and the breaking force was recorded in Newtons (N) using the digital display.

### 2.5. Hatching Traits

For the assessment of hatching traits, eggs were transported to a commercial hatchery. A total of 180 eggs were incubated in a multistage setter/incubator (Chick Master Multistage Classic Incubator, Jamesway Inc., 30 High Ridge Court, Cambridge, ON N1R 7L3, Canada) at 37.5–38 °C with 55% relative humidity for the first 18 days. Automatic turnings every 1–2 h (≥8 times/day) were applied to prevent the embryonic adhesion to the shell and to ensure uniform temperature distribution. Adequate ventilation was maintained to ensure oxygen supply and remove excess carbon dioxide. On day 18, eggs were transferred to a hatcher set at 36.1–36.9 °C and 65% relative humidity to prevent dehydration and support chick emergence. The hatching period was the final 3 days of the incubation (days 19–21) [18].

On the 30th day of trial, 10 eggs per replicate (60 per treatment) were used to determine the hatching traits across the treatments. The fertility of eggs was determined using the candling method on day 18 of incubation. Fertility (%) was calculated using the formula previously outlined as:

$$\mathrm{Fertility}\;(\%)\;=\;\left(\frac{\mathrm{Number}\;\mathrm{of}\;\mathrm{fertilized}\;\mathrm{eggs}}{\mathrm{Number}\;\mathrm{of}\;\mathrm{eggs}\;\mathrm{set}}\right)\times \;100$$

This parameter assesses the success of the artificial insemination process and the reproductive performance of the breeder flock. A break-out analysis was conducted to evaluate hatchability metrics. Hatchability (%) was calculated using the previously established formula.

$$\mathrm{Hatchability}\;(\%)=\left(\frac{\mathrm{Number}\;\mathrm{of}\;\mathrm{chicks}\;\mathrm{hatched}}{\mathrm{Number}\;\mathrm{of}\;\mathrm{fertile}\;\mathrm{eggs}}\right)\times 100$$

This parameter reflects the efficiency of the incubation process based on the proportion of fertile eggs that resulted in live chicks. Chicks were carefully taken out of the hatcher once they had hatched, and their vitality and health were also evaluated. Non-hatched eggs from each replicate were investigated after the experimental period to determine the reasons and timing of embryo death, categorized into early (1–7 days), intermediate (7–14 days), and late (14–21 days) mortality phases [28].

### 2.6. Tibial Breaking Strength

The halal slaughtering method (PS3733: 2016) was employed to slaughter the birds with a sharp knife. The jugular veins, carotid artery, trachea, and esophagus were cut during slaughter, and the neck of the chicken was properly cut till reaching the bone to ensure smooth bleeding. Tibia bones were selected as the representative bones to assess skeletal strength and bone quality due to their weight-bearing function, anatomical consistency, clinical relevance, and biomechanical properties. Tibial breaking strength was measured by using an electronic universal testing machine (Model WDW-100; Computer Control Electromechanical Universal Testing Machine, Jinan Hensgrand Instrument Co., Ltd., Jinan, China) [29,30,31]. Briefly, at the end of the experimental period (day 40), left tibia bones from 30 representative hens per treatment were collected through humane slaughter (PS3733: 2016). Bones were cleaned of soft tissue or debris, rinsed under running tap water, and gently scrubbed. They were soaked overnight in a mild detergent solution, followed by a second rinse and gentle brushing. The cleaned bones were air-dried at room temperature. Before testing, the proximal and distal condyles were removed using an electric cutter to ensure flat, uniform ends suitable for mounting. Care was taken to avoid damaging the bone shafts. Each tibia was vertically mounted and aligned in the machine to ensure axial loading along its longitudinal axis. The machine was calibrated, and parameters such as load rate and bone cross-sectional area were set. Increasing mechanical force was applied until the bone fractured. The maximum force at the point of fracture was recorded in Newtons (N). After each test, bone fragments were removed, and the equipment was cleaned before testing the next bone sample.

### 2.7. Mineral Analysis of Feed, Excreta, and Bone Samples

Mineral analysis of feed, bone, and excreta samples from each treatment was performed using inductively coupled plasma optical emission spectroscopy (ICP-OES) to analyze selected trace minerals, i.e., Zn, Mn, Cu, and Fe [32,33]. Briefly, the collection of excreta material from each replicate was done on the last three consecutive days of the trial. Then, samples of each day were mixed to form a representative sample for each replicate to perform mineral analysis. The right tibia bones from all the slaughtered birds were used for their mineral content estimation. The inductively coupled plasma machine (5110 ICP-OES, Agilent Technologies, Australia (M) Pty Ltd., 679 Springvale Road, Mulgrave, VIC, 3170 Australia), available at the Department of Environmental Sciences, PMAS-Arid Agriculture University Rawalpindi, Pakistan, was used for the analysis.

For sample preparation, all the feed, excreta, and bone samples were oven-dried. Then the samples were finely ground to pass through a 0.75-mm screen using a centrifugal mill (Ultra Centrifugal Mill ZM 200, Retsch, Haan, Germany, ring sieve size: 0.75 mm) before digestion. Wet digestion was performed using acid-based protocols previously established [34,35]. The digested samples were stored in capped tubes, and aliquots were transferred to labeled ICP vials for mineral analysis. For sample analysis, the ICP-OES instrument was calibrated using a standard solution (Agilent, Part # 5183-4687), and a dark current measurement was performed with a blank solution to verify wavelength calibration. Sample introduction was performed using the ASX-560 autosampler (Teledyne CETAC Technologies, Omaha, NE, USA),which aspirated and delivered the digested solutions into the plasma. Each sample was analyzed in triplicate to ensure precision and reproducibility.

The mineral elements were analyzed using the following wavelengths during ICP-OES analysis: Fe at 238.204 nm, Ni at 231.604 nm, Zn at 213.857 nm, Cr at 267.716 nm, Ca at 396.847 nm, K at 766.491 nm, Na at 589.592 nm, Cd at 214.439 nm, As at 188.980 nm, Mn at 257.610 nm, Pb at 220.353 nm, Co at 238.892 nm, Cu at 327.395 nm, Se at 196.026 nm, and P at 213.618 nm. Although a comprehensive analysis of all listed minerals was conducted, only those relevant to the study objectives, specifically Zn, Mn, Cu, Fe, and selected macro and microelements, were included in the final results. The spectral data obtained for each sample were interpreted to ensure accuracy, detect patterns across treatments, and derive meaningful conclusions regarding mineral bioavailability and excretion.

### 2.8. Statistical Analysis

Statistical analyses were performed using R Studio software version 2024.12.1 (Build 563) with R version 4.4.3. All collected data were initially assessed for normality and homogeneity of variance test using the Kolmogorov–Smirnov and Levene’s test, respectively. Subsequently, data were analyzed using one-way ANOVA; treatment means were compared using Fisher’s Least Significant Difference (LSD) test, with significance determined at *p* ≤ 0.05. The following mathematical model was applied:

Y_ij_ = μ + τ_i_ + ε_ij_

where

Y_ij_ = observation of the dependent variable recorded on the ith treatment group

μ = population mean

τ_i_ = effect of ith treatment (i = 1, 2, 3)

ε_ij_ = residual effect of jth observation on ith treatment, NID ∼ 0, σ^2^

## 3. Results

### 3.1. Egg Quality Parameters

Significant differences were observed among the treatments for certain egg quality traits, including eggshell breaking strength, egg shape index, yolk weight, and yolk diameter. Supplementation with organic glycinate minerals resulted in higher eggshell breaking strength compared to the inorganic minerals treatment (*p* ≤ 0.05; Table 3). Similarly, the egg shape index was higher (*p* ≤ 0.05) in organic treatment groups (Table 3). For internal egg quality, yolk weight was higher (*p* ≤ 0.05) in the OTM100 group as compared to the ITM100 group, while yolk diameter was greater (*p* ≤ 0.05) in the OTM50 group compared to the ITM100 group. However, no significant differences were found among the groups for eggshell thickness and shell weight (Table 4), as well as for egg weight, egg length, egg diameter, Haugh unit, yolk height, and albumen quality parameters.

### 3.2. Hatching Traits

This study also assessed the effects of replacing inorganic trace minerals with organic glycinate chelates on reproductive performance parameters, including fertility, hatchability, and embryo survival (Table 5). Hatchability was higher (*p* ≤ 0.05) in both organic mineral groups (OTM100 and OTM50) compared to the inorganic group (ITM100). Specifically, the hatchability was 10.78 and 12.23% higher in OTM100 and OMTM50 groups, respectively, compared to the ITM100. Although fertility did not differ among treatments (*p* > 0.05), OTM100 and OTM50 groups showed numerically higher fertility rates of 5.2 and 3.1% respectively, as compared to ITM100. No significant differences (*p* > 0.05) were observed among the groups for early and late embryonic mortality or dead-in-shells. However, mid-embryonic mortality was numerically higher (*p* ≤ 0.05) in the ITM100 group than in the OTM-treated groups (0.47 vs. 0 %).

### 3.3. Bone Quality Characteristics

When comparing the effects of inorganic trace minerals (ITM100) and organic glycinate trace minerals provided at two levels (OTM100 and OTM50), the OTM50 group showed higher (*p* ≤ 0.05) tibial breaking strength than the other two groups. The OTM100 group exhibited the lowest tibial breaking strength among all treatments. No significant differences (*p* > 0.05) were observed among treatment groups for tibia weight and tibial shaft area (Table 6).

### 3.4. Tibia Mineral Deposition

Regarding tibia mineral deposition, Cu content was higher (*p* ≤ 0.05) in the OTM100 group compared to the other treatments. However, no significant differences (*p* > 0.05) were observed among the groups for the other three minerals analyzed (Zn, Fe, Mn). However, Mn deposition was numerically lower in OTM50 compared with other groups, although differences were not statistically significant (*p* > 0.05) (Table 7).

### 3.5. Excreta Mineral Excretion

Significant differences (*p* ≤ 0.05) were observed in excreta Zn and Mn concentrations, with the ITM100 group exhibiting higher excretion levels compared to the OTM100 and OTM50 groups. In contrast, excreta concentrations of Cu, Fe, and cobalt (Co) did not differ (*p* > 0.05) among the treatments (Table 8).

## 4. Discussion

### 4.1. Egg Quality Characteristics

The higher eggshell breaking strength observed in birds supplemented with organic glycinate trace minerals can be attributed to their superior bioavailability, enhancing mineral incorporation into the shell matrix and resulting in stronger eggshells. By promoting the production of uronic acids and glycosaminoglycans within the shell membrane, which are essential for the formation of the palisade and mammillary layers, manganese (Mn) plays a crucial part in this process [36]. Additionally, Mn is a cofactor for the enzyme glycosyltransferase, which is necessary for the biomineralization of eggshells and implicated in the synthesis of proteoglycans [37]. Additionally, zinc plays a vital function in eggshell integrity because it is involved in carbonic anhydrase, which provides the carbonate ions needed for calcium carbonate deposition [38]. Egg weight and shell thickness can be increased by dietary zinc supplementation, according to a previous study by Sahin et al. [39].

In the current trial, birds fed organic glycinate minerals showed noticeably stronger eggshells than those fed inorganic minerals. Better shell quality provides more protection for the embryo and reduces breaking during handling and transportation, which increases hatchability. Economic losses from cracked and broken eggs have been estimated to be between 8% and 11% of total production [3]. Therefore, glycinate trace mineral supplementation can reduce egg breakage in the egg supply chain. These results are consistent with the study of Alfonso-Carrillo et al. [30], which found that laying hens at 52 to 60 weeks of age had similar eggshell strength when fed one-third of the necessary amounts of organic trace elements as opposed to full-dose inorganic minerals. Comparably, Chen et al. [40] showed that eggshells had greater strength at lower dosages of organic mineral premix (450 mg/kg) than at greater amounts (600 mg/kg) of both organic and inorganic sources. Ghasemi et al. [11] also observed that eggshell thickness in layers aged 32 to 50 weeks was independent of mineral source or dosage, which is in line with our findings.

The higher values of egg internal quality indicators, including yolk weight and diameter, may indicate enhanced nutrient delivery and liver function. Chelated minerals have been demonstrated to promote lipid metabolism [39] and hepatic activity [40], both of which are necessary for the production and deposition of yolk precursors such as lipoproteins and vitellogenin [41]. Additionally, their higher absorption promotes the use of calcium and magnesium, which are directly related to the creation and quality of shells [42]. These effects highlight the physiological advantages of chelated minerals, especially when aged layers are under a lot of production stress [43].

### 4.2. Hatching Traits

The current study observed a notable enhancement in hatchability in the organic glycinate trace mineral treatment groups compared to the inorganic trace mineral group. The finding of the current study was supported by Saber et al. [44], who explored the effects of using different proportions of inorganic and organic trace minerals in the diets of broiler breeders. They proposed that substituting inorganic trace minerals with either a half or full dosage of organic trace minerals in the diets of broiler breeders could improve hatching performance, growth, and carcass characteristics of their progeny. Chelated minerals may better bind vital nutrients like calcium and magnesium, allowing the body to absorb and use them more efficiently, which explains the higher hatchability observed with chelated mineral supplementation [45]. This improved bioavailability, as mentioned earlier, facilitates more efficient delivery of these nutrients [46], essential for eggshell formation [44], embryonic development [47], and overall hatchability [48]. Conversely, Umar Yaqoob et al. [19] determined that substituting inorganic trace elements with low-dose complexed glycinate minerals did not significantly affect laying performance or hatchability. The increased hatchability in this study indicates that organic glycinate trace minerals may help lower oxidative stress [49] and improve the general health of the breeding birds [50] both of which have a favorable impact on hatchability. The greater fertility of eggs can also be attributed to a greater utilization of the minerals involved in fertilization, such as Zn, Mn, Cu, and Se. These results are consistent with the findings of Londero et al. [51], where it was found that the addition of organic minerals to broiler breeder hen diets increased the fertility as compared with feeding the inorganic forms. Similarly, other studies have also demonstrated that the supplementation of laying hen diets with minerals in organic form improves hatching capacity from fertilized eggs [52], and fertility and hatching percentage [53]. Trace minerals such as zinc, manganese, and copper [54] play a crucial role in reproductive processes by participating in essential enzymatic reactions that enhance sperm quality [55], ovarian development [41], and hormonal regulation [56], thus promoting elevated fertility rates.

### 4.3. Tibia Quality Characteristics

The strength of bones is a measure of optimal skeletal health and must be preserved by a balanced diet. The OTM500 treatment group in this trial demonstrated higher tibial breaking strength than the ITM100 and OTM100 treatment groups, respectively. The elevated tibial breaking strength noted in the present study aligns with findings from Savaram Venkata et al. [57], who indicated that hens receiving 60% of the dosage from organic trace minerals exhibited greater tibial breaking strength compared to those receiving inorganic and organic trace minerals at commercially recommended levels. The increased tibial breaking strength at a lower dosage (50%) from an organic source depicts the higher bioavailability of minerals from an organic origin. Similar findings were reported by Manangi et al. [58], who demonstrated that the highest tibial strength was obtained in laying hens when Zn, Cu, and Mn were supplemented at 40, 10, and 40 ppm, respectively, from chelated sources. Zinc is essential for the function of osteoblasts and the production of collagen, the principal structural protein in bones [59]. Copper facilitates the cross-linking of collagen and elastin [60], which is essential for the elasticity of bone and structural integrity [61]. The production of the bone matrix and the control of bone mineralization depend on manganese [62]. Increased bioavailability of certain minerals from organic sources can improve these processes, resulting in stronger bones. Metallothionein (MT) sequesters excess trace minerals. In OTM100 and ITM100 treatments, excessive zinc and copper concentrations trigger metallothionein (MT) upregulation, leading to sequestration rather than utilization of these essential minerals. OTM50 prevents overstimulation of metallothionein function, allowing metallothionein to function within its homeostatic capacity, without leading MT to its detoxification role. Ultimately, chelated minerals stay available to be incorporated into metallo-enzymes, structural proteins, and lead to bone and eggshell mineralization. Trace minerals, particularly zinc, copper, and manganese, serve as cofactors for enzymes involved in collagen synthesis, bone mineralization, and matrix formation. The optimal dosage (50%) provides sufficient mineral availability for enzymatic function without triggering excessive metallothionein sequestration to reduce bioavailable mineral pools [63]. Moreover, the saturation of DMT1 (duodenal metal transporter-1) binding sites would lead to a competitive inhibition between zinc and copper transport. That explains why the OTM50 does not trigger excessive competition between minerals [63]. Therefore, our research trial suggests that adequate supplementation with organic glycinate trace minerals can lead to better bone health and potentially reduce the incidence of bone fractures and related issues in breeding birds.

### 4.4. Tibia Mineral Deposition

Tibial mineral content indicated mineral absorption and deposition in the skeletal system. The current study shows that zinc and Fe are not significantly altered in terms of deposition when supplemented from the two different sources. Copper deposition was similar when offered at full dose from an inorganic source and at a half dose from the organic glycinate source, implying that a half dose from an organic source has optimum bioavailability. We could not find differences in the tibial deposition of manganese. Likewise, Savaram Venkata et al. [57] reported that when inorganic trace minerals were replaced with organic trace minerals, at levels as low as 20% of the commercial recommendations, the Mn concentration in the tibia bone was not affected. However, a study on broilers by El-Husseiny et al. [64] demonstrated that the tibial content of Zn, Mn, Fe, and Cu was considerably reduced when these minerals were substituted with an organic source at half of the recommended mineral doses. The elevated copper deposition noted in the OTM100 group is likely due to the higher bioavailability of copper from glycinate chelates, which are absorbed more effectively owing to their shielding from antagonists and the utilization of amino acid transport mechanisms [4]. Copper is essential for oxidative defense, connective tissue integrity, and immunological function [65], indicating that enhanced tissue retention may be physiologically advantageous, especially in ageing breeder hens. The copper levels employed in this investigation adhered to breed standards and remained far below the maximum tolerated limits for chicken [66], hence demonstrating no risk of poisoning. Further investigations evaluating liver enzymes, oxidative stress indicators, and tissue residues over extended periods are necessary to delineate the safety margins and functional advantages of increased copper retention from organic sources. Despite supplementation differences, Zn, Fe, and Mn deposition remained stable, indicating homeostatic regulation of bone mineralization [67,68]. The role of Mn in cartilage and bone formation may make its tibia deposition less dependent on supplementation compared to Cu [68].

### 4.5. Excreta Mineral Excretion

Zinc and manganese excretion in the excreta material of breeding hens was lower in the groups supplemented with organic glycinate trace minerals compared to those supplemented with inorganic trace minerals. Similarly, copper was also less excreted in the organic glycinate trace mineral supplemented groups, although the difference was non-significant. As previously described, OTM50 does not offer a threshold or saturation level of minerals for the mineral transporters. The divalent metal transporter-1 (DMT1) exhibits finite transport capacity with saturable binding capacity. Therefore, higher absorption into the body. ITM100 and OTM100 lead to competitive inhibition between zinc, copper, and manganese, due to transporter saturation, for limited binding sites, and therefore, they end up in excreta [58]. Metallothionein sequesters excess trace minerals. Thus, higher sequestration and ultimately more mineral excretion through cellular sloughing of intestinal mucosa, leading to higher excreta concentrations of minerals in ITM100 and OTM100 treatments [57]. Conversely, the Fe and cobalt showed lower excreta concentration in the inorganic trace minerals supplemented groups than in the organic glycinate trace minerals supplemented groups. The lack of significant differences in tibial deposition of iron across all treatments suggests that, despite lower excretion, the absorbed iron is similarly utilized in both organic and inorganic forms.

Lower excretion of minerals in the excreta is in accordance with another study conducted by Wang et al. [69], which demonstrated that broilers fed with organic trace minerals at varying low levels of inclusion showed reduced excretion of Zn, Cu, Fe, and Mn as compared to the broilers fed on dietary inorganic trace minerals at commercially recommended levels. Similarly, Ramirez et al. [70] evaluated broiler performance using diets supplemented with inorganic trace minerals (Zn, Cu, Mn, Fe, Se, and I) at the Ross 308 recommended levels versus diets containing the same minerals from organic glycinate sources at one-third of those levels. They reported that birds fed inorganic trace minerals excreted higher amounts of Cu, Mn, and Zn in their litter compared with birds receiving the organic treatments. Lu et al. [71] also found that feeding organic trace minerals and coated trace minerals at lower levels of inclusion offered better mineral retention for Cu and Zn when compared with diets containing inorganic trace minerals, based on excreta mineral analysis. Consistent with our findings, Zhang et al. [36] conducted an experiment involving 57-week-old laying hens that were fed basal diets supplemented with commercial levels of inorganic trace elements and organic trace elements at inclusion levels of 20%, 30%, and 50%, in comparison to inorganic trace minerals. The groups supplemented with organic glycinate trace minerals had lower excreta mineral excretion than the group fed inorganic trace minerals.

Organic trace minerals remain stable and non-ionized before absorption; once they enter the digestive system, they can evade precipitation or adsorption by precipitants (such as phytic acid, phosphoric acid, and oxalic acid) within the intestinal tract [14]. Furthermore, proteinate minerals are conveyed and assimilated as amino acids; consequently, the primary advantage of employing organic minerals is linked to enhanced absorption, as they utilize the identical absorption pathways of the amino acids to which they are attached. This diminishes competition for inorganic trace mineral-binding sites and subsequently lessens the excretion of these minerals via bile and feces [72,73]. This enhancement of availability promotes growth, reproduction, immune system function, enzyme activity, and general health.

## 5. Conclusions

The administration of glycinate-based organic trace minerals during the late laying phase of commercial layer breeders enhanced eggshell quality, tibial breaking strength, fertility, and hatchability, while also enhancing mineral absorption and lowering mineral excretion via excreta relative to traditional inorganic sources. These findings highlight the practical advantages of utilizing organic trace minerals to enhance productive performance and nutrient efficiency, potentially aiding environmental sustainability by minimizing mineral waste. The enhancements in reproductive efficiency, egg quality, and mineral bioavailability may provide long-term economic and environmental benefits, particularly in high-value breeding enterprises. However, discrepancies with prior results and a limited comprehension of the underlying mechanisms necessitate additional investigation. Future research should consider dose–response trials, prolonged feeding periods, and molecular analyses of mineral transport mechanisms, gene expression, and bone metabolism to better understand trace mineral nutrition in layer breeder hens.

## Figures and Tables

**Table 1:** Experiment Design.

Sr. No.	Treatments	Description	Details
1	ITM100	Inorganic trace minerals at breed-recommended (Standard) levels	Treatments = 3 Replicates = 6 Experimental units = 18 Female Birds/Replicate = 9 Total Female Birds = 162 Male Birds = 18 Total Birds = 180 (Lohmann LSL-Ultralite White)
2	OTM100	Organic trace minerals at breed-recommended (Standard) levels	
3	OTM50	Organic trace minerals at half dose (50%) of recommended levels	

ITM100: Inorganic trace minerals at breed-recommended (Standard) levels; OTM100: Organic trace minerals at breed-recommended (Standard) levels; OTM50: Organic trace minerals at half dose (50%) of recommended levels.

**Table 2:** Ingredients and nutrient composition of diets (Kg/ton).

Ingredients	Diets		
	ITM100	OTM100	OTM50
Maize	677.2	677.2	677.2
Rice Polish	0.6	0.6	0.6
Soybean Meal	135	135	135
Canola meal	84	84	84
DL-Methionine	0.9	0.9	0.9
L-Lysine SO_4_	0.19	0.19	0.19
Limestone	94	94	94
MCP (Mono-Calcium Phosphate)	3.5	3.5	3.5
Salt	2	2	2
Sodium Bicarbonate	1	1	1
Phytase Enzyme ^1^	0.1	0.1	0.1
Vitamin Premix *	0.5	0.5	0.5
Inorganic Trace Minerals Premix *	1	--	--
Gly-Min-Blend *	--	1	0.5
**Total (kg)**	**1000**	**1000**	**1000**
**Dietary inclusion levels of selected trace minerals**			
Zn (mg/kg)	60	60	30
Mn (mg/kg)	100	100	50
Fe (mg/kg)	40	40	20
Cu (mg/kg)	10	10	5
**Analyzed Nutrients (% otherwise noted)**			
Crude Protein		18.2	
Metabolizable Energy (Calculated, Kcal/kg)		2.71	
Crude Fiber		4.45	
Crude Fat		4.2	
Dig. Lysine		0.67	
Dig. Methionine		0.33	
Calcium		4.2	
Available Phosphorus		0.35	
Sodium		0.16	
Chloride		0.14	

ITM100: Inorganic trace minerals at breed-recommended (Standard) levels; OTM100: Organic trace minerals at breed-recommended (Standard) levels; OTM50: Organic trace minerals at half dose (50%) of recommended levels. * Vitamin and trace mineral premixes were prepared to ensure the micronutrient requirements provided by the Lohmann LSL Parent stock guide for the late laying phase/phase 2 [18]. Glycinate minerals (E.C.O. Trace^®^, Biochem mbH, Oldenburg, Germany) were used in organic mineral diets. ^1^ The phytase enzyme (Quantum^®^ Blue, AB Vista Marlborough, Wiltshire, UK), an *E. coli* phytase having a minimum phytase activity of 5000 FTU/g enzyme, was added at 100 g/ton to obtain 0.15% more available P according to the manufacturer’s recommendations.

**Table 3:** Influence of mineral source (inorganic vs. organic glycinate) and inclusion level on egg quality metrics.

Parameters	ITM100	OTM100	OTM50	SEM	*p* Value
**Egg geometry**					
Egg weight (g)	59.8	59.3	58.9	0.68	0.49
Egg Length (mm)	57.5	57.7	56.9	0.46	0.25
Egg Breadth (mm)	44.1	43.4	43.7	0.49	0.38
Egg Shape Index	76.3 ^ab^	75.4 ^b^	76.8 ^a^	0.02	0.04 *
**Internal egg quality**					
Albumen Weight (g)	33.1	33.3	33.3	0.92	0.95
Albumen Height (mm)	8.45	8.43	8.58	0.33	0.89
Haugh Unit Score	91.2	91.6	91.1	1.65	0.96
Yolk Weight (g)	17.4 ^b^	18.4 ^a^	17.7 ^ab^	0.33	0.03 *
Yolk Diameter (mm)	42.1 ^b^	42.8 ^ab^	43.3 ^a^	0.36	0.02 *
Yolk Height (mm)	18.2	19.3	16.9	1.90	0.49
Yolk Index	43.4	46.2	39.2	4.61	0.34

* ^ab^—Letters indicate significant differences at the treatment level (*p* ≤ 0.05) using Fisher’s least significant difference test. Data are presented as Least Square Means with pooled Standard Error of the Mean, ITM100: Inorganic trace minerals at breed-recommended (Standard) levels; OTM100: Organic trace minerals at breed-recommended (Standard) levels; OTM50: Organic trace minerals at half dose (50%) of recommended levels.

**Table 4:** Influence of mineral source (inorganic vs. organic glycinate) and inclusion level on eggshell quality.

Egg Shell Properties	ITM100	OTM100	OTM50	SEM	*p* Value
Eggshell Thickness (mm)	0.39	0.42	0.44	0.02	0.10
Weight of Eggshell (g)	8.19	7.42	7.61	0.39	0.18
Eggshell Breaking Strength (N)	36.8 ^b^	43.5 ^a^	44.2 ^a^	2.69	0.03 *

* ^ab^—Letters indicate significant differences at the treatment level (*p* ≤ 0.05) using Fisher’s least significant difference test. Data are presented as Least Square Means with pooled Standard Error of the Mean, ITM100: Inorganic trace minerals at breed-recommended (Standard) levels; OTM100: Organic trace minerals at breed-recommended (Standard) levels; OTM50: Organic trace minerals at half dose (50%) of recommended levels.

**Table 5:** Impact of mineral source (inorganic vs. organic glycinate) and inclusion level on reproductive performance.

Parameters	ITM100	OTM100	OTM50	SEM	*p* Value
Fertility (%)	80.1	84.5	82.7	2.31	0.59
Hatchability of set eggs (%)	60.0 ^b^	67.5 ^a^	66.2 ^a^	1.10	0.01 *
Hatchability of fertile eggs (%)	63.9 ^b^	71.6 ^a^	72.1 ^a^	1.81	0.01 *
Dead in Shell (%)	11.3	9.85	9.10	0.71	0.56
Early Embryonic Death (%)	5.12	6.40	6.50	0.70	0.54
Mid Embryonic Death (%)	0.47 ^a^	0.00 ^b^	0.00 ^b^	0.05	0.04 *
Late Embryonic Death (%)	1.25	2.40	1.75	0.26	0.42
Pipped Out Dead (%)	2.00	1.05	0.00	0.31	0.07

* ^ab^—Letters indicate significant differences at the treatment level (*p* ≤ 0.05) using Fisher’s least significant difference test. Data are presented as Least Square Means with pooled Standard Error of the Mean, ITM100: Inorganic trace minerals at breed-recommended (Standard) levels; OTM100: Organic trace minerals at breed-recommended (Standard) levels; OTM50: Organic trace minerals at half dose (50%) of recommended levels.

**Table 6:** Effect of mineral source (inorganic vs. organic glycinate) and inclusion level on tibia bone quality.

Parameters	ITM100	OTM100	OTM50	SEM	*p* Value
Tibia Weight (g)	6.97	6.71	6.47	0.25	0.20
Tibial Shaft Area (mm^2^)	36.6	37.2	34.7	1.48	0.25
Tibial Breaking Strength (MPa)	29.6 ^b^	27.9 ^b^	39.0 ^a^	0.51	0.01 *

* ^ab^—Letters indicate significant differences at the treatment level (*p* ≤ 0.05) using Fisher’s least significant difference test. Data are presented as Least Square Means with pooled Standard Error of the Mean, ITM100: Inorganic trace minerals at breed-recommended (Standard) levels; OTM100: Organic trace minerals at breed-recommended (Standard) levels; OTM50: Organic trace minerals at half dose (50%) of recommended levels.

**Table 7:** Influence of mineral source (inorganic vs. organic glycinate) and inclusion level on trace mineral content in the tibia.

Parameters	ITM100	OTM100	OTM50	SEM	*p* Value
Tibia Zn (ppm)	1.96	1.87	1.74	0.24	0.68
Tibia Cu (ppm)	0.06 ^b^	0.14 ^a^	0.05 ^b^	0.02	0.02 *
Tibia Fe (ppm)	57.5	57.7	56.9	0.46	0.25
Tibia Mn (ppm)	0.12	0.13	0.09	0.01	0.28

* ^ab^—Letters indicate significant differences at the treatment level (*p* ≤ 0.05) using Fisher’s least significant difference test. Data are presented as Least Square Means with pooled Standard Error of the Mean, ITM100: Inorganic trace minerals at breed-recommended (Standard) levels; OTM100: Organic trace minerals at breed-recommended (Standard) levels; OTM50: Organic trace minerals at half dose (50%) of recommended levels.

**Table 8:** Effects of mineral source (inorganic vs. organic glycinate) and inclusion level on fecal excretion of trace minerals.

Parameters	ITM100	OTM100	OTM50	SEM	*p* Value
Fecal Zn (µg/g)	661.0 ^a^	425.3 ^b^	430.3 ^b^	37.8	0.01 *
Fecal Mn (µg/g)	479.0 ^a^	211.3 ^b^	233.0 ^b^	15.7	<0.01 *
Fecal Cu (µg/g)	62.0	53.3	52.3	4.38	0.13
Fecal Fe (µg/g)	56.3	73.0	67.7	24.0	0.79
Fecal Co (µg/g)	39.4	39.6	40.8	0.87	0.28

* ^ab^—Letters indicate significant differences at treatment level (*p* ≤ 0.05) using Fisher’s least significant difference test. Data are presented as Least Square Means with pooled Standard Error of the Mean, ITM100: Inorganic trace minerals at breed-recommended (Standard) levels; OTM100: Organic trace minerals at breed-recommended (Standard) levels; OTM50: Organic trace minerals at half dose (50%) of recommended levels.

## Data Availability

The original contributions presented in this study are included in the article. Further inquiries can be directed to the corresponding authors.
